# Genetic Models for the Study of Luteinizing Hormone Receptor Function

**DOI:** 10.3389/fendo.2015.00152

**Published:** 2015-09-29

**Authors:** Prema Narayan

**Affiliations:** ^1^Department of Physiology, School of Medicine, Southern Illinois University, Carbondale, IL, USA

**Keywords:** luteinizing hormone receptor, gonadotropins, inactivating and activating mutations, genetic models, knockout mice, knockin mice, transgenic mice

## Abstract

The luteinizing hormone/chorionic gonadotropin receptor (LHCGR) is essential for fertility in men and women. LHCGR binds luteinizing hormone (LH) as well as the highly homologous chorionic gonadotropin. Signaling from LHCGR is required for steroidogenesis and gametogenesis in males and females and for sexual differentiation in the male. The importance of LHCGR in reproductive physiology is underscored by the large number of naturally occurring inactivating and activating mutations in the receptor that result in reproductive disorders. Consequently, several genetically modified mouse models have been developed for the study of LHCGR function. They include targeted deletion of LH and LHCGR that mimic inactivating mutations in hormone and receptor, expression of a constitutively active mutant in LHCGR that mimics activating mutations associated with familial male-limited precocious puberty and transgenic models of LH and hCG overexpression. This review summarizes the salient findings from these models and their utility in understanding the physiological and pathological consequences of loss and gain of function in LHCGR signaling.

## Introduction

The luteinizing hormone/chorionic gonadotropin receptor (LHCGR), together with the glycoprotein hormone receptors, follicle-stimulating hormone receptor (FSHR) and thyroid stimulating hormone receptor (TSHR), belongs to the G protein-coupled receptor superfamily (1). LHCGR is the target receptor for the pituitary-derived luteinizing hormone (LH) and the highly homologous placental chorionic gonadotropin (CG). The fully processed human LHCGR is 675 amino acid residues long and is characterized by a large extracellular domain that is sufficient for hormone binding (2). The LHCGR has 11 exons with the first 10 exons encoding the extracellular domain and exon 11 encoding the C-terminal tail of the hinge region of the extracellular domain, the transmembrane helices with the connecting extra- and intracellular loops, and the cytoplasmic tail (1, 2). Functional LHCGR is essential for sex differentiation in the fetus and reproductive function in the adult. During fetal development in males, LHCGR present in the interstitial fetal Leydig cells of the testis binds to placental CG to produce testosterone required for male sexual differentiation (3, 4). Postnatally, LH stimulates LHCGR in the Leydig cells to produce testosterone required for development of puberty, male secondary sexual characteristics, and spermatogenesis. Female fetal sex differentiation does not require LH or steroid hormones. Postnatally, in females, LHCGR is present in the theca cells lining the follicle, mature granulosa cells, stromal cells, and luteinized cells. LH activation of LHCGR in the theca cells stimulates androgen production, thereby providing the substrate for conversion to estradiol by follicle-stimulating hormone (FSH) induced aromatase in granulosa cells and triggering puberty (5). LHCGR activation is required for ovulation and subsequent progesterone production by the corpus luteum (6). The canonical signaling pathway mediated by LHCGR for steroidogenesis is the Gαs/cAMP/protein kinase A pathway. However, LHCGR can also activate additional pathways, including the Gαq/inositol phosphate/protein kinase C, protein kinase B, and ERK1/2 pathways (6–10). In the testis, the ERK1/2 cascade modulates androgen synthesis as well as proliferation/survival of Leydig cells (11–15). In the ovary, LHCGR-mediated activation of the EGF network and ERK1/2 cascade via Gαs/cAMP is required for resumption of meiosis, cumulus expansion, and luteinization, whereas follicular rupture is dependent on both Gαs and Gαq/11 (6, 16).

## Naturally Occurring Mutations in *LHCGR* and *LHB* Genes

The large number of naturally occurring mutations and polymorphisms in the LHCGR gene that result in disorders of sexual development and reproductive function (4, 17) highlights the critical role of this receptor in reproduction. Mutations are inactivating, resulting in loss of receptor function, or activating resulting in constitutive activation of the receptor. These mutations have been particularly useful in elucidating the molecular mechanisms of LHCGR activation. Inactivating mutations are present in all domains of the receptor and may be missense mutations, insertions, deletions, and nonsense mutations. As a result, there may be partial inactivation or complete loss of receptor function caused by premature truncation of the receptor protein or failure to traffic to the cell surface (18). The mutations are recessive (19) and patients are either homozygous or compound heterozygous carriers. In males, inactivating mutations result in failure of testicular Leydig cell differentiation, resulting in the disorder called Leydig cell hypoplasia (LCH). Two types of Leydig cell hypoplasia are identified. The severe form is caused by mutations that result in loss of receptor protein, failure of receptor to traffic to cell surface, or failure to transduce a signal. This results in 46,XY male pseudohermaphroditism with female external genitalia, undescended testes, low testosterone, and high LH levels. The milder form, caused by mutations that allow partial LHR function, results in micropenis and hypospadia (4, 17, 20). Testicular histology showed hyalinized basement membrane in the seminiferous tubules with Sertoli cells but few or no germ cells (4). Females with inactivating mutations exhibit amenorrhea and infertility, but normal feminization at puberty indicating that LH is not essential for pubertal development. Activating mutations resulting in single amino acid replacements in LHCGR were the first to be described in patients with familial male-limited precocious puberty (FMPP) (21, 22). This is a rare disorder affecting upto 9/million (Orphanet/NIH, Office of Rare Diseases). In early studies, before the availability of molecular analyses, this disorder was called familial testotoxicosis (23, 24). These mutations are heterozygous and inherited in an autosomal dominant male-limited pattern although a few sporadic cases have been reported (25). Clinically, these boys present with precocious puberty by 3–4 years of age, Leydig cell hyperplasia, and high circulating levels of testosterone in the context of prepubertal levels of LH (26–28). Surprisingly, female carriers of activating mutations are normal. The mutations are limited to exon 11 and clustered in transmembrane helix 6 and the third intracellular loop with aspartic acid at position 578 most commonly mutated to glycine (D578G) (22, 28). This mutation is found in about 62% of all FMPP cases and 29% of all sporadic cases of male-limited precocious puberty (29). Only one activating somatic mutation (D578H) has been identified so far in boys with precocious puberty and Leydig cell adenomas (30–32) and this mutation has not been identified in boys with FMPP.

In contrast to the large number of activating and inactivating mutations in LHCGR, no germ line mutations in the common α-subunit or hCGβ subunits and no gain-of-function mutations in LHβ have been identified. Only three inactivating mutations in LHβ resulting in complete loss of bioactive LH have been reported in four men and one woman (33–35). In all cases, the males were normally masculinized at birth but later presented with delayed or lack of spontaneous puberty, hypogonadism, low testosterone levels, and infertility. Testicular biopsy revealed absence of complete spermatogenesis and mature Leydig cells (33, 34). This suggests that LH is not required for male sexual differentiation. Fetal testosterone production begins autonomously and then becomes dependent on maternal hCG activation of LHCGR. Postnatal testicular development and function is, however, dependent on pituitary LH. Treatment with exogenous LH and hCG resulted in an increase in testosterone, indicating that receptor function was normal (33). The single female patient showed normal pubertal development but presented with secondary amenorrhea and infertility (35). The normal pubertal development is similar to that seen in women with a homozygous inactivating mutation in the LHCGR gene (36–39). A fourth mutation resulting in a deletion of amino acid residues 10–12 of LHβ was reported in a man and his sister (40). In spite of undetectable levels of LH and low serum and intratesticular testosterone, the man had complete spermatogenesis and normal sperm count. The low residual activity of the mutant LH detected *in vitro* was apparently sufficient for normal spermatogenesis. The single female patient underwent normal puberty, but developed secondary amenorrhea and infertility (35).

## Genetic Models to Study LHCGR Function

Several mouse models have been developed that model human reproductive disorders involving LHCGR signaling. They include knockout models of LH and LHCGR to mimic the inactivating LHβ and LHCGR mutations (41–43) and knockin mice expressing a constitutively active mutant LHCGR (44, 45) to mimic the activating LHCGR mutations. In addition, several transgenic models of enhanced LH/hCG action have been generated. They include mice expressing the LHβ-CGβ carboxyl terminal peptide (CTP) fusion protein under the control of the bovine common α subunit promoter (46), mice expressing hCG under the control of the ubiquitin C (47, 48) or metallothionein promoter (49), and mice expressing a yoked hCG–LHCGR fusion protein and D556H rat LHCGR under the control of the inhibin α subunit promoter (50).

### LHβ knockout mice

#### Phenotype of Male Mice

LHβ knockout mice were generated by deleting the coding sequence of the *Lhb* gene (43). Heterozygous mice were fertile and homozyous male and female mice were infertile. Serum levels of LH were undetectable, while serum FSH levels were normal. Male mice had significantly smaller testes and accessory glands consistent with reduced levels of serum and testicular testosterone. Testes contained very few Leydig cells, which were mostly fetal and immature adult Leydig cells as indicated by increased levels of serum androstenedione and upregulation of the fetal Leydig cell marker, thrombospondin. Spermatogenesis in the mutant mice was arrested at the round spermatid stage, indicating that LH and/or testosterone are required for the last step in spermatogenesis. Some Sertoli cell markers (FSHR) and inhibin α were unchanged in the knockout mice, while others (anti-mullerian hormone and the inhibin β subunits) were upregulated, indicating that lack of LHβ caused both somatic and germ cell defects. Sexual differentiation and fetal gonadal development was normal in the knockout mice, indicating that pituitary LH is not required for fetal testosterone production and gonadal development. A similar result was seen in mice lacking the common alpha subunit (α-GSU) for gonadotropin hormones (51).

#### Phenotype of Female Mice

Female KO mice were also infertile with abnormal estrous cycles. Serum estradiol and progesterone were greatly reduced. Primary and secondary follicles were present in the ovary, but healthy antral, preovulatory, and corpora lutea (CL) were absent. Antral follicles contained degenerating oocytes. However, the theca cell layer appeared normal, indicating that the differentiation of this layer was independent of LH signaling. Expression of steroidogenic enzyme genes was reduced in both sexes consistent with the reduction in steroid hormone levels. Treatment of the knockout mice with hCG rescued the phenotype in both male and female mice, indicating that receptor responsiveness was normal.

### LHCGR knockout mice (LuRKO)

Two groups independently reported the development of the LHCGR knockout mice by deleting part of the promoter region and exon 1 (41) or exon 11 encoding the transmembrane and intracellular domain of the receptor (42). Both models showed a complete loss of functional receptor resulting in infertility in both sexes. The reproductive phenotypes described by the two groups were similar.

#### Phenotype of Male Mice

Sexual differentiation was normal, demonstrating again that, unlike in humans, fetal testosterone production required for masculinization is gonadotropin independent in mice, as previously shown with the LHβ and the common alpha subunit (αGSU) knockout models (43, 51). The mice were phenotypically normal at birth. Postnatally, the mice exhibited cryptorchidism, reduced testis size, poorly developed accessory sex glands, and micropenis. Testosterone levels were dramatically reduced while levels of both serum LH and FSH were increased. The testis had dramatically reduced Leydig cell numbers and spermatogenesis was arrested in the round spermatid stage (41, 42). The slightly elevated FSH levels in the LuRKO mice apparently stimulated spermatogenesis to the round spermatid stage. Further analysis revealed that the testicular histology of the LuRKO mice was similar to wild type (WT) mice until about 3 weeks of age. After 3 weeks, the growth rate of the testis was dramatically decreased (52) and the interstitium lacked adult-type Leydig cells. Leydig cell-specific and steroidogenic enzyme genes showed similar level of expression in the neonatal WT and LuRKO mice but the LuRKO did not show the pubertal increase seen in WT mice. Testicular testosterone levels were similar between the genotypes at birth. Expression of the fetal Leydig cell marker *Tsp2* was similar between the genotypes but the adult Leydig cell markers, *Hsd3b6* and *Hsd17b3*, were downregulated at postpubertal ages. Together, these data suggested that testosterone production by fetal Leydig cells and initial differentiation of the adult Leydig cell population are not dependent on LHCGR action. However, differentiation to the mature Leydig cells with steroidogenic potential requires LHCGR signaling.

Testosterone replacement therapy at puberty restored full spermatogenesis and testicular descent, but failed to restore adult-type Leydig cells (53, 54), indicating that androgen-independent actions of LH are required for adult Leydig cell differentiation. However, the fertility of the mice could not be completely restored (54, 55). The subfertile phenotype was determined to be due to reduced epididymal sperm counts and low ejaculatory frequency. Additionally, inflammation in the prostate and vas deferens was observed (54). Interestingly, additional studies by Zhang et al. (56) reveal that complete spermatogenesis with the appearance of elongated spermatids can be observed in the LuRKO mice at 12 months of age although intratesticular testosterone levels remained suppressed and similar to the levels in 2-month-old mice. This result suggested that the low level of constitutively produced intratesticular testosterone was sufficient for differentiation of round to elongated spermatids.

#### Phenotype of Female Mice

Female KO mice were phenotypically normal at birth, which is not surprising since female sexual differentiation is independent of ovarian function and ovarian LHCGR expression begins after birth (57, 58). The age of vaginal opening was delayed in the KO mice and they did not exhibit normal estrous cyclicity. The uterus was atrophic with a thinning of all layers and lack of endometrial glands. Serum levels of estradiol and progesterone were suppressed but not absent (41). Estradiol and progesterone replacement therapy of 4-week-old KO mice for a period of 3 weeks stimulated vaginal growth and increased uterine size (55). However, the number of endometrial glands remained low and fertility was not restored. The ovaries were greatly reduced in size and ovaries contained preantral and antral follicles but no preovulatory follicles or CL (41, 42). This indicates that both ovulation and the maturation of antral to preovulatory follicles require LH action. The requirement of LHCGR action for follicle maturation beyond the antral stage was novel and was further investigated in the LuRKO mice (59). This study showed that progression of folliculogenesis beyond the antral stage and induction of ovulation could not be achieved by hCG or recombinant FSH in the absence of LHCGR.

### Knockin mice expressing the constitutively active mutant D582G LHCGR (KiLHR^D582G^)

We have recently generated mice expressing a constitutively active Asp582Gly (D582G) mutant in the mouse LHCGR (44). The mice are heterozygous with one WT allele replaced by the mutant allele as seen in patients with FMPP. Expression from both WT and mutant alleles could be detected in the testis. This mutation is analogous to the most prevalent Asp578G mutation in humans with FMPP. When tested in cell culture, the mouse D582G LHCGR showed a similar binding affinity as WT LHCGR. However, the basal level of cAMP was 23-fold higher in cells expressing the mutant receptor compared to WT. These levels are much higher than the three- to fourfold increase in basal cAMP seen with the human LHCGR (22, 60), and similar to that obtained with the D578H mutation found in Leydig cell adenomas (30).

#### Phenotype of Male Mice

**Figure 1:**
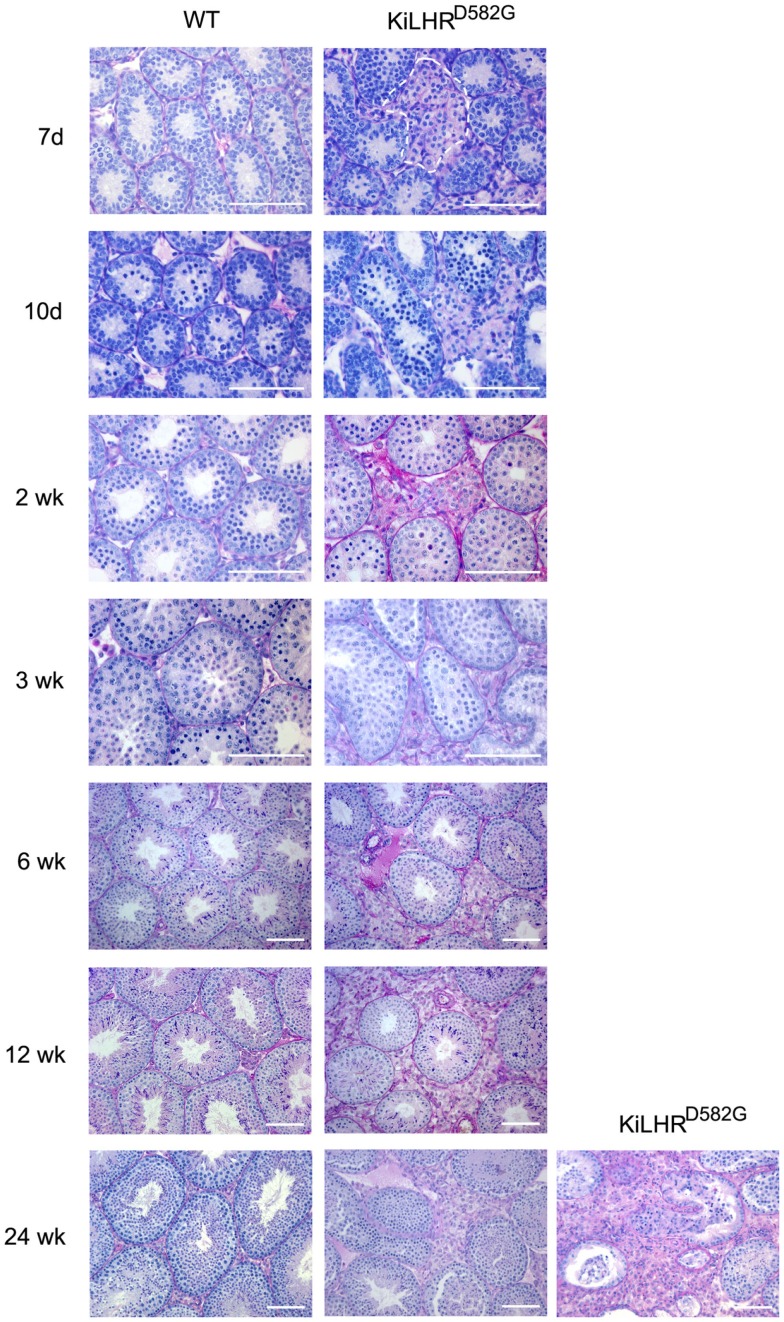
**Leydig cell hyperplasia in KiLHR^D582G^ mice**. Representative photomicrographs of PASH stained testis sections of WT and KiLHR^D582G^ from 7 days to 24 weeks of age. At least three animals per age and genotype were examined. The Leydig cell interstitium is marked by the dashed lines in sections from the 7-day-old mice. Sections of testis from two 24-week-old KiLHR^D582G^ mice shows variation in the severity of the Leydig cell hyperplasia. Bar = 100 μm. From McGee and Narayan (44).

KiLHR^D582G^ mice exhibited precocious puberty as shown by the advancement of balanopreputial separation and the early detection of mouse urinary proteins in the urine by 15 days compared to 22 days for WT mice (44). Both are androgen-dependent events and indicators of puberty in mice (61, 62). Testosterone levels were elevated as early as 7 days of age while gonadotropin levels were suppressed. The high testosterone levels resulted in enlarged seminal vesicles and prostate but not in significantly higher body weights. Several of the Leydig cell-specific genes involved in the steroidogenic pathway, including *Lhcgr* were upregulated. In spite of the precocious puberty, no advancement in the timing of spermatogenesis was seen. Spermatogenesis and Sertoli cell development and function appeared unaffected although testis size was decreased in the KiLHR^D582G^ mice presumably due to the suppressed FSH levels. Significantly, Leydig cell hyperplasia was detected as early as 7 days (Figure 1). The hyperplasia was patchy with a higher prevalence around the periphery of the testis. Precocious maturation of adult Leydig cells occurred in the mutant mice leading to the hyperplasia. The severity of the hyperplasia appeared to increase in the older animals (Figure 1). Interestingly, the KiLHR^D582G^ mice became progressively infertile and were unable to produce litter after an average age of 5–6 months. This was not due to a defect in spermatogenesis as the number of total and motile sperm from the cauda epididymis of 6-month-old KiLHR^D582G^ mice was not different from the WT mice.

#### Phenotype of Female Mice

**Figure 2:**
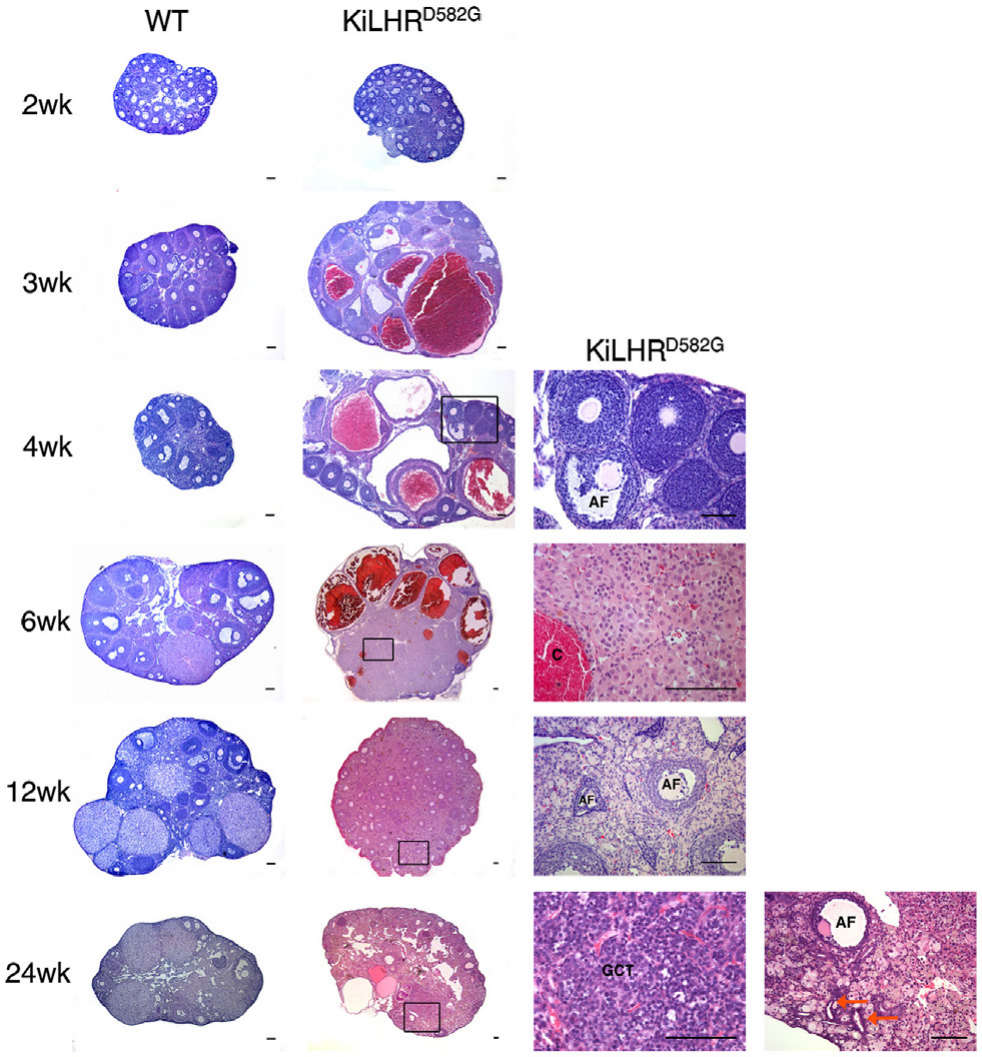
**Ovarian pathology in KiLHR^D582G^ mice**. Representative photomicrographs of H&E stained ovary sections of WT and KiLHR^D582G^ mice. At least three animals per age and genotype were examined. Higher magnification of the boxed areas in sections of KiLHR^D582G^ mice at 4, 6, 12, and 24 weeks are shown in the last column. C, hemorrhagic cyst; GCT, granulosa cell tumor; AF, atretic follicle. Arrow indicates tubulostromal hyperplasia. Scale bars represent 100 μm. From Hai et al. (45).

Female KiLHR^D582G^ mice exhibited precocious puberty and the age of vaginal opening was advanced by 2 weeks compared to WT mice (45). Mutant mice demonstrated irregular estrous cyclicity and were infertile. A temporal study from 2 to 24 weeks of age demonstrated elevated levels of androstenedione, testosterone, estradiol, and progesterone in the serum of KiLHR^D582G^ mice compared to WT mice. Consequently, gonadotropin levels were suppressed. The ovaries and uterus of the KiLHR^D582G^ mice were enlarged and large cysts were apparent in the gross morphology of the ovaries. The ovarian histology was normal in the 2-week-old KiLHR^D582G^ mice. However, degenerating follicles and hemorrhagic cysts were observed starting at 3 weeks of age (Figure 2). Follicles did not progress beyond the preantral stage likely due to lack of FSH stimulation. CL were not present indicating anovulation. Extensive stromal cell hypertrophy and hyperplasia with luteinization was apparent. In 6-month-old mice, granulosa cell tumors were evident in 50% of the KiLHR^D582G^ mice. Interestingly, the anovulatory phenotype could not be rescued by superovulation with pregnant mare serum gonadotropin (PMSG) and hCG. Although preovulatory follicles with oocytes could be detected in the ovaries of the KiLHR^D582G^ mice, they did not rupture to form CL. This result suggests that neither the WT or D582G mutant LHCGR is able to respond to exogenous gonadotropins. LH-dependent induction of Gαs/cAMP is required for the activation of the EGF network and the ERK1/2 cascade responsible for oocyte maturation and cumulus expansion while LH activation of Gαq/11 and Gαs is required for ovulation (6, 16). Coexpression of a constitutively active hLHR (L457R) that was unresponsive to additional hormone stimulation, with the WT LHR in cell culture, caused an attenuation of the hCG/Gαs stimulated cAMP production by WT LHCGR (63). This attenuation was not due to a decrease in the cell surface expression of the WT receptor, but due to the activation of phosphodiesterase 4D3 resulting in decreased levels of cAMP. Perhaps a similar mechanism occurs *in vivo* in the granulosa cells of the KiLHR^D582G^ mice to inhibit ovulation. The body weights of KiLHR^D582G^ mice were higher than that of WT mice. However, there were no changes in body fat composition or insulin resistance as seen in experimentally induced hyperandrogenic rodent models of polycystic ovarian syndrome (PCOS).

### LHβ overexpressing transgenic mice

Transgenic mice expressing a chimeric protein (bLHβ-CTP) consisting of the bovine LHβ subunit fused in frame to the C-terminal peptide of hCGβ subunit driven by the pituitary-specific bovine glycoprotein α-subunit (α-GSU) promoter was first reported by Risma et al. (46). The addition of the CTP increased the circulatory half-life of LH resulting in constitutive high levels (5- to 10-fold) in the female but not male mice. Preliminary observations with the male mice showed reduced fertility and smaller testis in spite of normal hormone levels and the mice were not characterized further. Female LHβCTP mice presented with precocious puberty, elevated levels of testosterone, and estradiol and infertility due to anovulation (64). The predominant ovarian phenotype was the presence of hemorrhagic cysts with widespread luteinization of the interstitial tissue. Accelerated depletion of primordial follicles was also observed (65). Although anovulation could be reversed by treatment with exogenous gonadotropins, pregnancy could not be maintained due to defects in uterine receptivity and mid-gestation pregnancy failure (66). Additional ovarian defects included granulosa cell tumors by 5 months of age only in the genetic background of CF-1 mice (67). In a hybrid background, the phenotype resembled the luteoma of pregnancy and it was shown that three genes likely control the different phenotypes (67). In addition to the ovarian tumors, the LHβCTP mice also developed mammary gland tumors (68). An interesting non-reproductive phenotype resulting from the elevated LH in the LHβCTP mice is adrenal hyperplasia and induction of LHCGR expression and activity in the adrenal gland. As a result, corticosterone production is stimulated (69). This phenotype was dependent on the dysfunctional ovaries of the transgenic mice as gonadectomy abolished the adrenocortical hyperfunction. LHβCTP mice also have elevated levels of prolactin caused by the enhanced estrogen synthesis (69). It has been suggested that prolactin synergizes with LH in the induction of LHCGR expression in the adrenal glands. Additionally, LHβCTP female mice are obese (70). Obesity was associated with hyperphagia, increased intra-abdominal fat, increased levels of serum leptin and insulin, and reduced thermogenic activity of brown adipose tissue. The elevated androgens and corticosterone most likely contribute to the obesity seen in the LHβCTP females as ovariectomy normalized corticosterone levels and reversed the obesity and hyperphagia (70). Transgenic females also developed renal abnormalities, including enlarged bladders, dilated ureters, and hydronephrosis presumably due to the elevated steroids (46).

### hCG overexpressing mice

Additional models of enhanced LH/hCG action were indepen­dently developed by two groups. Supraphysiological levels of hCG were expressed using the human ubiquitin C promoter or the mouse metallothionein-1 (MT-1) promoter (47–49). Transgenic mice expressing only the hCGβ subunit or both the common α subunit and hCGβ from multiple tissues were examined in both sexes.

#### Phenotype of Male Mice

In male mice expressing only the hCGβ subunit (hCGβ^+^) under the human ubiquitin C promoter, circulating levels of dimeric hCG were detected, indicating that the hCGβ subunit associated with the endogenously produced α subunit in the pituitary (48). However, dimeric hCG levels were only 3-4 fold higher than WT mice because the amount of endogenous α−GSU produced by the pituitary is rate-limiting. These mice were fertile and presented with only a mild reproductive phenotype of smaller testes (48). By contrast, mice expressing the hCGβ subunit under the mouse MT-1 promoter were infertile although hCG dimer could not be detected in the serum of these mice and the testes were morphologically and physiologically normal (49). This was surprising as individual subunits of hCG are devoid of activity (71).

Mice expressing both subunits (hCGαβ^+^) under the control of the ubiquitin C promoter produced extremely high levels of dimeric hCG with about 2000-fold increase in circulating LH/hCG bioactivity in male mice (48). Serum and testicular testosterone and progesterone were elevated in spite of down regulation of receptor expression. Male mice were infertile and vaginal plugs were not observed when mice were mated with superovulated females in spite of motile and morphologically normal sperm in the cauda epididymis. Adult mice at 2–6 months of age showed smaller testes with normal tubular structure. However, progressive degenerative changes in the seminiferous tubules were observed. Mice developed focal Leydig cell hyperplasia/hypertrophy but not adenomas. Subsequent studies in prepubertal mice showed Leydig cell adenomas that were of fetal Leydig cell origin and disappeared at puberty (72). No sign of precocious puberty was observed in these young mice in spite of elevated levels of testosterone. The seminal vesicle and prostate were enlarged. The distention and sperm accumulation in the distal vas deferens as well as the dilated urinary bladder and ureters and enlarged kidneys pointed to a functional uretheral obstruction caused by the overproduction of secretory fluids or impaired emptying of the accessory glands. This may be a likely cause of infertility in these mice. Aggressive behavior of the males toward the females during mating may also contribute to the infertile phenotype.

Matzuk et al. used the MT-1 promoter to generate transgenic mice expressing low and high levels of the dimeric hCG (49). Males with low levels of heterodimer expression showed progressive infertility of unknown etiology. Adult males with high expression levels had similar reproductive defects as described above, including reduced testis size, Leydig cell hyperplasia, enlarged fluid-filled seminal vesicles, elevated testosterone levels, and infertility. Males were very aggressive toward both transgenic and non-transgenic males or females.

#### Phenotype of Female Mice

In contrast to male mice, female transgenic hCGβ^+^ mice using the ubiquitin C promoter associated with the endogenously expressed mouse α-subunit to produce a 40-fold increase in bioactive LH/hCG compared to WT females (47). Although female hCGαβ^+^ mice expressing both subunits as transgenes produced a 2000-fold elevation in bioactive LH/hCG, the phenotype of the hCGβ^+^ and hCGαβ^+^ mice were similar. Mice presented with precocious puberty, disrupted estrous cycles, and infertility. Adult mice were obese due to abdominal fat accumulation. Transiently elevated estradiol and persistent elevation of testosterone and progesterone were observed in the transgenic mice. The ovaries were significantly enlarged with massive luteinization, resembling luteomas, which may explain the transient increase in estradiol. Hemorrhagic cysts and CL were present. Females also developed macroprolactinomas and mammary gland tumors at 9–12 months of age. Serum prolactin was greatly elevated and may help maintain the ovarian luteinization and progesterone production. Metastasis of the mammary tumors to the liver, spleen, and lung was seen in about 47% of the mice. The mammary gland and pituitary tumors were dependent of ovarian function and ovariectomy prevented their development even when hCG levels were high. Subsequent studies (73) showed that the hCGαβ^+^ mice with the 2000-fold elevation in bioactive LH/hCG develop teratomas.

Mice overexpressing only the hCGβ subunit under the MT-1 promoter were infertile although heterodimer could not be detected in the serum (49). These mice also had ovarian defects, including block in folliculogenesis and cysts. Mice with low levels of hCG dimer became progressively infertile. Mice expressing high levels of hCG dimer had elevated estradiol levels and developed enlarged cystic and hemorrhagic ovaries with stromal cell proliferation and enlarged thecal cell layers. Degenerating kidneys were also evident. These mice did not develop mammary gland or pituitary tumors.

### Yoked hormone receptor and rat D556H LHCGR transgenic mice

Transgenic mice expressing a yoked hormone receptor (YHR^+^) genetically engineered by covalently linking hCG to LHCGR was generated to create a model for constitutively active LHCGR mutations. In cell culture, YHR exhibited an increase in the basal level of both cAMP and inositol phosphate similar to that seen with constitutively active mutants (50, 74). The transgene was expressed under the control of the gonadal-specific inhibin α-subunit promoter and male mice were fertile (75). Serum and testicular testosterone levels were elevated in YHR^+^ mice at prepubertal ages of 3 and 5 weeks, but not at 8 and 12 weeks of age. Consistent with the increased testosterone was the increase in seminal vesicle weights. However, there was no evidence of precocious puberty. Serum levels of LH and FSH were decreased due to elevated testosterone. Testis size was decreased at all ages in YHR^+^ mice and histological analysis showed a significant decrease in the cross-sectional area of the tubules. Spermatogenesis was not initiated earlier and Leydig cell hyperplasia, as seen in patients with constitutively active LHCGR, was also not observed. Further characterization of the male phenotype showed a reduction in the number of Leydig cells in YHR^+^ mice accompanied by a reduction in the expression of several Leydig cell specific genes (76). The difference in the phenotype of the YHR^+^ mice from humans with activating LHCGR mutations is likely because the inhibin α-subunit promoter did not faithfully mimic the spatial and temporal expression of LHCGR.

Female YHR^+^ mice exhibited precocious puberty and were subfertile. Increased levels of estradiol and progesterone were observed at 5 weeks of age. However, the hormonal changes were no longer apparent in adult mice. Increased folliculogenesis and CL were observed in 5-week-old mice and interstitial cell hypertrophy, degenerating follicles, and follicular cysts were observed in adult mice.

Transgenic mice expressing the rat D556H LHCGR under the control of the inhibin α-subunit promoter was also generated (50). This mutation corresponds to the somatic D578H mutation found in Leydig cell adenomas (30–32). Unfortunately, male and female mice expressing the transgene were infertile which prevented their further characterization. Preliminary analysis of two infertile founder male mice did not show testicular adenomas.

## Comparison of the Mouse Models with Human Reproductive Pathologies of LHCGR Function

### Inactivating mutations and knockout models

The mouse models of inactivation of LHCGR function have verified existing knowledge, but more importantly have provided new information on the function of LH. Although the homozygous inactivating mutations in the *LHB* gene result in single amino acid, changes they cause result complete loss of bioactivity similar to the deletion of the *Lhb* gene in the mouse. The phenotype of the homozygous LHβ knockout male and female mice closely mimics that of humans with the inactivating LHβ mutations (Table 1). In males, sexual differentiation is normal in humans and mice. However, in humans, testosterone required for masculinization *in utero* is dependent on placental hCG stimulating LHCGR while in mice it is independent of LH action. LH is critical postnatally and both humans and mice show hypogonadism, low testosterone levels, arrest of spermatogenesis, Leydig cell hypoplasia, and infertility.

**Table 1:** **Summary of genetic models for the study of LHCGR function**.

Gene (mutation/mouse model)	Major human phenotypes	Major mouse phenotypes	Reference
**A. COMPARISON OF HUMAN AND MOUSE PHENOTYPES FOR INACTIVATING AND ACTIVATING MUTATIONS IN LH AND LHCGR**			
*LHB* (inactivating/knockout)	Male: infertility, delayed puberty, hypogonadism, Leydig cell hypoplasia, spermatogenic arrest, normal sexual differentiation	Male: infertility, hypogonadism, Leydig cell hypoplasia, spermatogenesis arrested at round spermatid stage, normal sexual differentiation	(43)
	Female: normal pubertal development, normal uterus, folliculogenesis blocked at antral stage, secondary amenorrhea, infertility	Female: hypogonadal, folliculogenesis blocked at antral stage, hypoplastic uterus, infertility	
*LHCGR* (inactivating/knockout)	Male: micropenis, hypospadia, pseudohermaphroditism, Leydig cell hypoplasia, germ cell defects	Male: infertility, Leydig cell hypoplasia, underdeveloped sex organs, spermatogenesis arrested at round spermatid stage, normal sexual differentiation	(41, 42)
	Female: normal pubertal development, amenorrhea, folliculogenesis blocked at antral stage, infertility	Female: delayed puberty, underdeveloped accessory glands, follicles arrested at antral stage, infertility	
*LHCGR* (activating/knockin)	Male: precocious puberty, Leydig cell hyperplasia, high testosterone	Male: precocious puberty, Leydig cell hyperplasia, high testosterone, progressive infertility	(44, 45)
	Female: normal	Female: precocious puberty, cystic hemorrhagic ovaries with stromal cell hyperplasia with luteinization, granulosa cell tumors, infertility	

**Construct**	**Major phenotypes**	**Reference**	
**B. TRANSGENIC MOUSE MODELS OF ENHANCED LH/hCG ACTION**			
α-GSU promoter/bLHβ-CTP	Males: subfertility, smaller testis	(46, 66, 67)	
	Females: precocious puberty, infertility, polycystic ovaries, stromal cell luteinization, granulosa cell tumors, mammary gland tumors, hydronephrosis		
Ubiquitin C promoter/hCGβ	Males: no phenotype	(47, 48)	
	Females: precocious puberty, infertility, luteinized cystic ovaries, prolactinomas, mammary gland tumors		
Ubiquitin C promoter/hCGαβ	Males: infertility, adult Leydig cell hyperplasia, fetal Leydig cell adenomas, urethral obstruction, and kidney defects	(48, 73)	
	Females: infertility, ovarian teratomas		
MT-1 promoter/hCGβ	Males: infertility	(49)	
	Females: infertility, cystic, and hemorrhagic ovaries		
MT-1 promoter/hCGαβ	Males: infertility, Leydig cell hyperplasia	(49)	
	Females: infertility, cystic, and hemorrhagic ovaries, degenerating kidneys		
Inhibin α-subunit promoter/YHR	Males: fertile, elevated testosterone with smaller testis, and Leydig cell hypoplasia	(75)	
	Females: subfertile, precocious puberty, interstitial cell hypertrophy		

Female LHβ knockout mice displayed several characteristics similar to the single female patient with LHβ inactivating mutation identified thus far. Folliculogenesis was arrested at the antral stage resulting in infertility. Surprisingly, this patient had normal puberty, with normal sized ovaries and breast and uterine development. Presumably, the low level of estradiol was sufficient for normal breast and uterine development (35).

Inactivating mutations in LHCGR in human males have a more severe phenotype, resulting from abnormal sexual differentiation, that those in LHβ emphasizing the importance of LHCGR signaling in sexual development and postnatal pubertal development. By contrast, the LuRKO mouse demonstrates that LHCGR signaling is not essential for mouse sexual differentiation, highlighting a major species difference. LHCGR activation is, however, important for postnatal development in both species. The phenotypes of the LHβ and LHCGR knockout male mice are very similar. Spermatogenic arrest at the round spermatid stage in both models indicates that FSH alone is not sufficient for full spermatogenesis and that testosterone produced by LH signaling is required for postmeiotic germ cell maturation. A novel observation that resulted from studies on the LHCGR knockout mice was that at 12 months of age, qualitatively complete spermatogenesis was possible in the absence of LH stimulated high testosterone production (56). This process, however, requires a long priming period. From a clinical standpoint, this finding may explain why men treated with testosterone for contraceptive purposes are not azoospermic (56).

Women with inactivating LHCGR and LHβ mutations have normal pubertal development, indicating that LH signaling is not essential for puberty in women. This process is more dependent on FSH signaling as demonstrated by lack of pubertal development in women with inactivating mutations in the FSHβ or FSHR (77). LH signaling is, however, required in the mouse where absence of LHCGR delays pubertal development (Table 1). There is a remarkable similarity in the ovarian phenotype of women with inactivating LHβ and LHCGR mutations, LHβ knockout and LuRKO mice showing a block in folliculogenesis at the antral stage. The studies from these mouse models clearly showed for the first time that, in addition to its well-known role in ovulation, LH is also required for the final stage of follicular maturation before ovulation. Studies with the LuRKO mice (59) also clarified that ovulation could not be induced by FSH in the absence of functional LHCGR as has been previously suggested (78, 79).

### Activating mutations and knockin models

The most common mutation in FMPP is the D578G mutation in transmembrane helix 6. In the mouse model (KiLHR^D582G^) generated in our laboratory, the corresponding D582G mutation was introduced into the WT *Lhcgr* gene (44). The male mouse is a good phenocopy of men with constitutively activating LHCGR mutations as shown by the development of precocious puberty, Leydig cell hyperplasia and high testosterone (Table 1). A major difference between the mouse model and FMPP patients is that spermatogenesis is not advanced in the KiLHR^D582G^ mouse. Presumably, the seminiferous cycle of 35 days is at a minimum in mice and cannot be shortened further even with premature testosterone production. Furthermore, spermatogenic development requires the expression of the androgen receptor in Sertoli cells and significant levels are not detected till around postnatal day 15 in mice (80). It has been demonstrated that premature expression of the androgen receptor in Sertoli cells can accelerate spermatogenic development (80). Two new findings form the mouse model, not previously reported or confirmed in FMPP cases, are that the hyperplasia is not uniform throughout the testis and that it results from the precocious development of adult Leydig cells. The progressive infertility and hyperplasia seen in the KiLHR^D582G^ mice suggest that FMPP patients may be susceptible to infertility and perhaps tumor development later in life. There is only one report of a FMPP patient with the D578G mutation who was diagnosed with nodular Leydig cell hyperplasia (81), primarily due to lack of long-term follow-up of FMPP patients past puberty.

The phenotype of female KiLHR^D582G^ mice (45) is distinctly different from women with activating mutations who are normal (82–84). Mice undergo precocious puberty and are infertile with significant ovarian pathology of hemorrhagic cysts, stromal cell hyperplasia, and granulosa cell tumors. The reason for this discrepancy is unclear. The low level of LHCGR expression in prepubertal girls, the requirement for the activation of both LHCGR and FSHR for puberty, and less efficient androgen synthesis in theca cells compared to Leydig cells (4, 82). A higher magnitude of LHCGR activation may be required for development of ovarian pathology. In this context, the mouse LHCGR has a higher level of constitutive activation than the human receptor and the phenotype of the female KiLHR^D582G^ mice is similar to transgenic models of LH and hCG overexpression. A novel finding from the study of KiLHR^D582G^ mice is the dominant negative effect of the mutant receptor on the function of the WT receptor. The lack of rescue of the anovulatory phenotype by the administration of PMSG and hCG indicated that D582G LHCGR inhibits signaling of the WT receptor and is the first report of such an effect *in vivo*.

In general, the knockout and knockin mouse models are close phenocopies of the human disorders; however, species differences in LHCGR function clearly exist. Although these differences provide useful knowledge on LHCGR physiology, they should be considered when the mice are used as models of human diseases.

### Transgenic models of enhanced LH/hCG action

Activating mutations in LH or hCG have not been identified. However, there are physiological and pathological states when these hormone levels are elevated. hCG is produced in high amounts in the first trimester of pregnancy and in gestational trophoblastic disease (1). In men and women, hCGβ, hyperglycosylated hCGβ, and occasionally hCG dimer are secreted from a variety of tumors (1). Gonadotroph adenomas induce high gonadotropin levels and hypersecretion of LH is observed in pathological conditions such as PCOS. Chronic elevation of gonadotropins occurs in menopause and this is proposed as risk factor for ovarian cancer (85).

A comparison of the phenotypes of the overexpressing LH/hCG mice is shown in Table 1. The LHβ-CTP model was the first overexpressing model described. Hormone levels were not elevated in male mice because the α-GSU promoter is inefficient in the male. Overexpression of dimer hCG driven by either the ubiquitin C or MT promoter resulted in similar male phenotypes of infertility and Leydig cell hyperplasia with high testosterone levels. Aggressive behavior of the males toward the females was observed in both hCG models and this may contribute in part to the infertile phenotype. The infertility in the ubiquitin C promoter-driven hCG mice was not due to defects in sperm but possibly due to the urethral obstruction (48).

The pathological changes seen in the female LHβ-CTP and hCG overexpressing mice were similar with precocious puberty and infertility. Significant pathology was seen in all models, with cystic, hemorrhagic, and luteinized ovaries. Granulosa cell tumors were seen in the LHβ-CTP mice, but ubiquitin C promoter-hCG mice had ovarian luteomas and teratomas and no tumors were found in the MT-hCG mice. Both LHβ-CTP and ubiquitin C promoter-hCG mice develop mammary gland tumors, but the latter also develop prolactinomas in older mice. This indicates that chronic high levels of gonadotropins promote tumor formation in gonadal and non-gonadal tissues. The pituitary and mammary gland tumors are secondary effects of aberrant gonadal function. The inhibin α-subunit promoter-YHR mice did not exhibit the robust changes seen in the overexpressing mice in either males or females.

The LH/hCG overexpressing models and the inhibin α-subunit promoter-YHR mice were expected to mimic the activating LHCGR mutations by precocious activation of the receptor. Comparison of the male KiLHR^D582G^ mice with the transgenic mouse models of LH/hCG overexpression showed similar phenotypes of Leydig cell hyperplasia and high testosterone. Similar to the ubiquitin C promoter-hCG mice, the progressive infertility in the KiLHR^D582G^ mice was not due to sperm defects. However, there were distinct differences as well. The overexpressing models did not exhibit precocious puberty and Leydig cell adenomas in the ubiquitin promoter-hCG mice were of fetal origin rather than from adult Leydig cells as seen in the KiLHR^D582G^ mice. The inhibin α-subunit promoter-YHR mice did not exhibit phenotypes similar to either the KiLHR^D582G^ mice or LH/hCG overexpressing mice except for the increase in testosterone. Presumably, the promoter used was regulated differently from the endogenous LHCGR.

The reproductive (precocious puberty, infertility) and ovarian phenotypes (cystic, hemorrhagic ovaries, interstitial cell hypertrophy with luteinization, granulosa cell tumors) of the KiLHR^D582G^ mice are similar to the LH/hCG overexpressing mice. The luteomas, teratomas (47), and enlarged thecal cell layer are specific to the hCG overexpressing mice (49, 73). The extra-gonadal phenotypes of pituitary and mammary gland tumors are not also seen in the KiLHR^D582G^ mice. The obese phenotype seen in the LHβ-CTP and ubiquitin-hCG overexpressing mice is not evident in the KiLHR^D582G^ mice. In contrast to the KiLHR^D582G^ mice, the anovulatory phenotype of the LHβ-CTP could be rescued by exogenous gonadotropins (66), further confirming an inhibition of WT LHCGR function in the KiLHR^D582G^ mice. The differences in the phenotypes between the overexpressing models and KiLHR^D582G^ mice, particularly the extra-gonadal phenotypes, are likely due to the high levels of LH/hCG secreted by transgenes that were expressed ubiquitously under the control of promoters that do not mimic the spatial or temporal expression of LHCGR. Some of the differences between the three overexpressing models may be the result of different levels of hormone production, different promoters, and genetic background. However, the remarkable number of similarities between the models emphasizes the importance of LH/LHCGR action on reproductive physiology and pathophysiology.

## Future Perspectives

Mouse models are now available that can mimic the genetic alterations in LH and LHCGR and physiological and pathological states of hormone excess. These models have reinforced the well-established roles of LH and LHCGR but have also uncovered novel functions. The KiLHR^D582G^ mice can be used to investigate the long-term reproductive and non-reproductive abnormalities that result from constitutive LHCGR activity, particularly the mechanism of the progressive infertility. This information will be useful in predicting the long-term health of FMPP patients. This mouse model will be valuable to test new therapeutic agents that can block constitutive activity and to further determine the *in vivo* mechanism of the dominant negative effect of the mutant receptor on WT receptor function. Based on the phenotypic changes seen in the models of enhanced LH/hCG action, it appears that female physiology is more sensitive to changes in LH-mediated signaling. In particular, these models can be used to better understand the signaling mechanisms important in the development of ovarian and extra-gonadal tumors and understand the role of LH in obesity and related metabolic changes. The models can be helpful in sorting out the controversies and conflicting data regarding the extra-gonadal actions of LHCGR. In general, all the described models are amenable to large-scale gene expression profiling to better understand LHCGR signaling mechanisms.

An area that has not been explored extensively is the neurological changes associated with LHCGR signaling. Testosterone is important in brain development and sexual differentiation. Behavioral studies have shown that FMPP patients are susceptible to attention deficit hyperactivity disorder and a higher rate of anxiety disorder (86). The KiLHR^D582G^ mice are an ideal animal model to assess cognitive and behavioral changes associated with FMPP. There is also increasing evidence that elevated levels of LH can exacerbate age-related cognitive decline in Alzheimer’s disease (87, 88). LHβ-CTP mice exhibit cognitive deficits (89). Considering that functional LHCGR is expressed in the brain (90), it will be of interest to determine if cognitive and behavioral changes are due to direct LHCGR signaling in the brain or indirectly due to its activity in gonads. These mechanisms and the contributions of direct vs. indirect effects on the brain can be teased out with the mouse models. Clearly, these mouse models have the potential to uncover novel aspects of LHCGR signaling.

## Conflict of Interest Statement

The author declares that the research was conducted in the absence of any commercial or financial relationships that could be construed as a potential conflict of interest.
